# 585. Effects of Legislation Restricting Reproductive and LGBT+ Rights on Infectious Disease Fellowship Match Rates

**DOI:** 10.1093/ofid/ofae631.180

**Published:** 2025-01-29

**Authors:** Michael Z Chen, Alan L Hutchison, Aniruddha Hazra, Anna Czapar

**Affiliations:** University of Chicago, Chicago, IL; University of Chicago Medicine, Chicago, Illinois; University of Chicago, Chicago, IL; University of Chicago, Chicago, IL

## Abstract

**Background:**

Over the last decade, infectious disease (ID) fellowship match rates have declined despite increasing workforce need. Many factors may influence rates, including effects of local political climate. Since 2022, many states have restricted reproductive and/or LGBT+ rights. As 43% of ID physicians identified as women in 2021 (AMA) and 6% of graduating medical students entering medicine residencies identified as lesbian, gay, or bisexual from 2016-2019 (Mori, 2021), there is a need to examine impact of local politics on match rates.

**Table 1.**
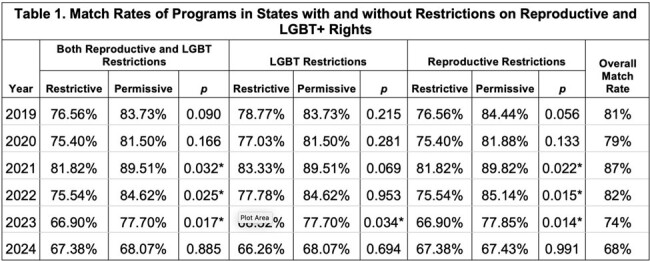
Match Rates of Programs in States with and without Restrictions on Reproductive and LGBT+ Rights

**Methods:**

Match rates for ID fellowship programs from 2019-2024 were tabulated by state based on publicly available NRMP data. States were categorized as reproductively-restrictive if they enacted laws limiting abortion and/or LGBT+ restrictive if they enacted laws limiting gender expression, LGBT+ healthcare or curriculum after 2022 based on reporting from the NYTimes and the Trevor Project. States without any laws were categorized as permissive. Differences in match rates in states with both restrictions, one restriction, or no restrictions were compared using two-sample z-tests of proportion.

**Figure:**
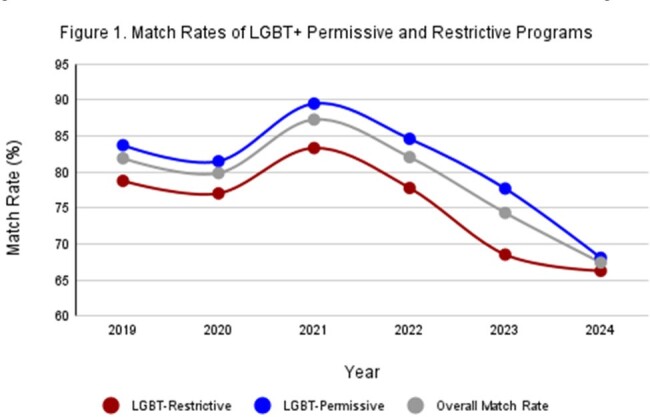

**Results:**

From 2019-2024, overall ID fellowship match rates declined from 81% to 68%, but permissive states (*n* = 121) consistently had higher match rates than restrictive states (*n* = 76) (Table 1). Match rates were significantly higher in LGBT-permissive states than restrictive ones in 2021 (*p* = .034) (Fig 1), in reproductively-permissive states than restrictive ones from 2021-2023 (*p* = .022, .015, .014) (Fig 2), and in states without any restrictions than states with both restrictions from 2021-2023 (*p* = .032, .025, .017) (Fig 3). There were no significant differences in match rates when comparing programs based on restrictions in 2019, 2020, and 2024.

**Figure:**
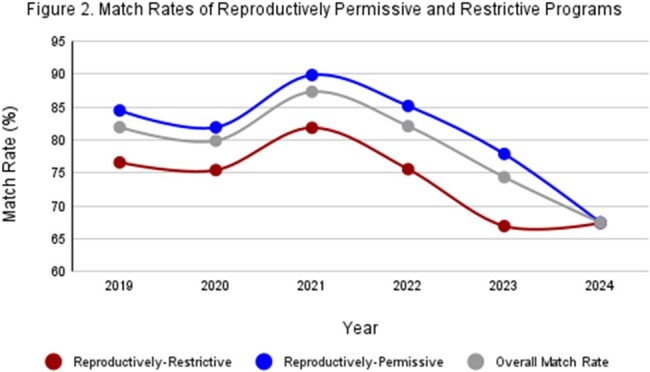

**Conclusion:**

Significant differences in match rates from 2021-2023 between programs in states with and without restrictions of rights suggests that political climate may have a measurable impact. However, all match rates converged at their lowest in 2024 regardless of program location. This may indicate a basal match rate across states, and political climate may influence match rates above this level. Longitudinal study of application volumes and match rates and surveys to assess applicant attitudes are warranted.

**Figure:**
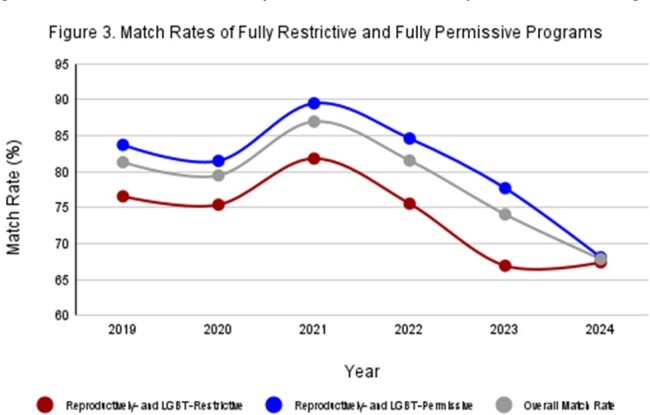

**Disclosures:**

**Aniruddha Hazra, MD**, Gilead Sciences: Advisor/Consultant|Gilead Sciences: Grant/Research Support|ViiV Healthcare: Advisor/Consultant

